# Deviant reporter expression and P2X4 passenger gene overexpression in the soluble EGFP BAC transgenic P2X7 reporter mouse model

**DOI:** 10.1038/s41598-020-76428-0

**Published:** 2020-11-16

**Authors:** Antonio Ramírez-Fernández, Lidia Urbina-Treviño, Giorgia Conte, Mariana Alves, Björn Rissiek, Anna Durner, Nicolas Scalbert, Jiong Zhang, Tim Magnus, Friedrich Koch-Nolte, Nikolaus Plesnila, Jan M. Deussing, Tobias Engel, Robin Kopp, Annette Nicke

**Affiliations:** 1 Walther Straub Institute of Pharmacology and Toxicology, Faculty of Medicine, LMU Munich, Munich, Germany; 2 Max Planck Institute of Psychiatry, Molecular Neurogenetics, Munich, Germany; 3 Department of Physiology and Medical Physics, Royal College of Surgeons in Ireland, Dublin, D02 YN77 Ireland; 4 Department of Neurology, University Medical Center Hamburg-Eppendorf, Hamburg, Germany; 5 Institute of Immunology, University Medical Center Hamburg-Eppendorf, Hamburg, Germany; 6 Institute for Stroke and Dementia Research, Munich University Hospital, LMU Munich, Germany; 7 FutureNeuro, Science Foundation Ireland Research Centre for Chronic and Rare Neurological Diseases, Royal College of Surgeons in Ireland, Dublin, D02 YN77 Ireland; 8 Munich Cluster of Systems Neurology (Synergy), Munich, Germany

**Keywords:** Biological techniques, Molecular biology, Neuroscience

## Abstract

The ATP-gated P2X7 receptor is highly expressed in microglia and has been involved in diverse brain diseases. P2X7 effects were also described in neurons and astrocytes but its localisation and function in these cell types has been challenging to demonstrate in situ. BAC transgenic mouse lines have greatly advanced neuroscience research and two BAC-transgenic P2X7 reporter mouse models exist in which either a soluble EGFP (sEGFP) or an EGFP-tagged P2X7 receptor (P2X7-EGFP) is expressed under the control of a BAC-derived *P2rx7* promoter. Here we evaluate both mouse models and find striking differences in both P2X expression levels and EGFP reporter expression patterns. Most remarkably, the sEGFP model overexpresses a P2X4 passenger gene and sEGFP shows clear neuronal localisation but appears to be absent in microglia. Preliminary functional analysis in a *status epilepticus* model suggests functional consequences of the observed P2X receptor overexpression. In summary, an aberrant EGFP reporter pattern and possible effects of P2X4 and/or P2X7 protein overexpression need to be considered when working with this model. We further discuss reasons for the observed differences and possible caveats in BAC transgenic approaches.

## Introduction

The P2X7 receptor plays a key role in inflammation^1,2^ and is considered a therapeutic target for a variety of diseases including depression, Alzheimer’s disease, and epilepsy^2–7^. While there exists a general consent regarding its presence and proinflammatory functions in macrophages and microglia, its pathophysiological roles in neurons and astrocytes are less clear. Although P2X7-mediated effects on neurotransmitter release, neuronal excitability, and neuronal cell death have been shown^8–10^, its expression and function in neurons have been particularly challenging to demonstrate^11,12^. This is partly due to its complex pharmacology and a lack of specific antibodies. To allow cell type-specific visualization and analysis of protein expression in complex tissues, the generation of bacterial artificial chromosome (BAC) transgenic reporter mice has proven to represent an invaluable method that greatly advanced neuroscience research^13–15^. In this approach, reporter proteins, such as enhanced green fluorescent protein (EGFP), are either directly expressed under the control of BAC-derived regulatory sequences of the gene of interest or a BAC-controlled Cre recombinase is used to cell-specifically drive fluorescent protein expression in floxed reporter mouse lines. Although numerous studies proved that this approach is able to reliably reproduce the expression pattern of the respective gene^16–18^, several caveats need to be considered: (i) the stability and regulation of the resulting reporter mRNA and protein might differ from that of the gene of interest, (ii) critical regulatory elements (which are generally not known) may be absent in the chosen BAC, (iii) possible position effects caused by the random integration of the modified BAC in the genome should be excluded by comparison of several transgenic mouse lines, and (iv) integration of multiple fluorescent reporter gene copies is usually required to allow efficient visualization.

**Figure 1:**
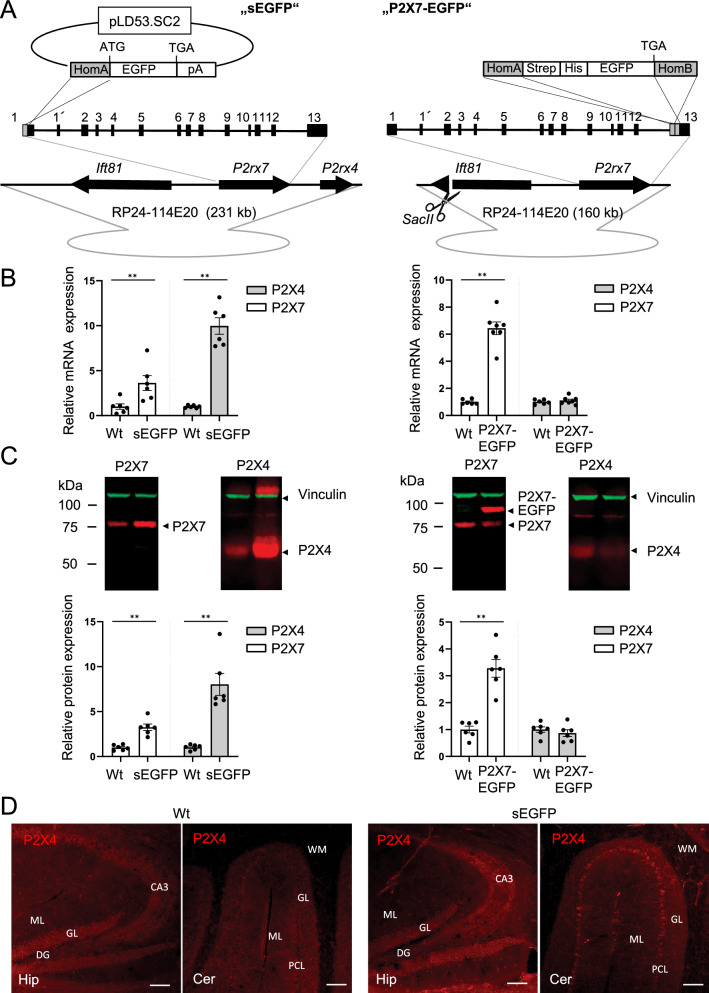
Comparison of BAC transgenic P2X7 reporter mice and *P2rx4* and *P2rx7* expression in these mice. (**A**) The sEGFP sequence followed by a poly(A) signal was inserted in the start ATG in exon 1 of the *P2rx7* gene. The recombination strategy resulted in co-integration of the pLD53.SC2 vector downstream of the poly(A) signal. In case of the P2X7-EGFP fusion construct, a Strep-His-linker followed by the EGFP sequence was inserted in front of the Stop TGA in exon 13 of *P2rx7*. Use of two homology arms prevented vector integration. Intraflagellar transport protein *Ift81* and, in case of the sEGFP model, *P2rx4* are introduced as passenger genes together *with P2rx7.* In the P2X7-EGFP model, *Ift81* is destroyed by *Sac II* linearization. HomA/B: gene-specific homology arms; opened grey circle: vector backbone of the BAC. For further details see Table 1. (**B**) Quantitative RT-PCR analysis of hippocampal *P2rx4* and *P2rx7 expression* in both mouse models compared to wt mice. Data were normalized to expression of β-actin and the respective levels in wt controls. Bars represent mean ± SEM from 2 independent experiments and 6–7 mice (both sexes, around eight weeks of age). Significance was analysed using the Mann–Whitney test. **p  < 0.01. (**C**) Representative Western blot analysis of P2X4 and P2X7 protein expression. 50 µg of total lung protein (extracted in 1% NP-40) were loaded per lane. Proteins were immunoblotted and quantified by infrared imaging with fluorescent secondary antibodies. Bars represent mean ± SEM from two independent experiments and 6 mice (both sexes, 10–35 weeks of age). Significance was analysed using the Mann–Whitney test. **p  < 0.01. Note that the overexpression of P2X4 leads to aggregation and multimer formation in the sEGFP sample. The full gels are shown in Supplementary Fig. 6. (**D**) Immunofluorescence staining with a P2X4 antibody to demonstrate increased P2X4 expression level in the sEGFP reporter mouse. Three different animals were analysed per group, and representative images of cerebellum and hippocampus are shown. GL, granular cell layer; ML, molecular cell layer; DG, dentate gyrus; PCL, Purkinje cell layer; WM, white matter; CA3, cornu amonis region 3; Scale bars 100 µm.

In the case of P2X7, two BAC-transgenic reporter mouse models have been generated (Fig. 1A). In the Tg(P2rx7-EGFP)FY174Gsat mouse (https://www.gensat.org/), a sequence encoding a soluble EGFP (sEGFP) followed by a polyadenylation signal (pA) was inserted into Exon 1 of the *P2rx7* gene^13,19^, which should prevent its expression. This mouse has been available for more than ten years and has been frequently used as a reference for *P2rx7* expression in the brain and as tool to monitor its expression in in vitro studies and disease models^20–23^. More recently, the Tg(RP24-114E20P2X7451P-StrepHis-EGFP)Ani reporter model was described. Here, the EGFP sequence was integrated in frame into the last exon of the *P2rx7* gene resulting in the expression of a P2X7-EGFP fusion protein^24^.

Although in both P2X7 reporter models the EGFP constructs are expected to be expressed under the control of the *P2rx7* promoter, comparison of the available data indicated substantial differences in their cell type-specific expression^24^. Consequently, in the present study we carefully compared the RNA and protein expression of P2X7 and its neighbouring gene, the P2X4 receptor, in both mouse models. Furthermore, we performed a detailed comparison of the cell type-specific mRNA and protein expression in the CNS. Our results raise serious concerns regarding the reliability of the regional and cell type-specific reporter expression in the sEGFP reporter mouse model. Furthermore, initial functional experiments in a model of *status epilepticus* suggest that overexpression of P2X4 and/or P2X7 influence the pathophysiological response in this mouse.

## Results

### Comparison of BAC constructs

A critical point in generation of BAC transgenic mice is the selection of an optimal BAC clone: if the 5´and 3´ non-translated regions are too short, critical regulatory sequences might be missing. If these regions are too long (> 200,000 bp^13^), engineering becomes less efficient and neighbouring passenger genes could be included and cause unwanted effects. As shown in Table 1 and Fig. 1A, different BAC clones were used in the two reporter mouse models. In both, the gene encoding the intraflagellar transport protein (IFT)81 is present upstream of the *P2rx7* gene. IFT81 is part of the core of the IFT-B complex, which plays a crucial role in cilia formation and has been associated with ciliopathies^25,26^. In the sEGFP but not in the P2X7-EGFP reporter model, the *P2rx4* gene that lies downstream of the *P2rx7* gene is also present. Both passenger genes should theoretically result in the overexpression of their gene products. However, in the P2X7-EGFP model, the *Ift81* gene was cleaved prior to transformation using a singular *SacII* restriction site. Overexpression of the *Ift81* gene product in the sEGFP model remains to be determined.

**Table 1:** Comparison of BAC clones used for P2X7 reporter mouse lines and genes contained in one or both of the clones (data according to ensembl database).

	Begin	End	Length (bp)
RP23-181F3	122,505,388	122,736,196	230,809
RP24-114E20	122,541,108	122,701,415	160,308
*Ift81*	122,550,204	122,614,518	64,314
*P2rx7*	122,643,911	122,691,432	47,521
*P2rx4*	122,707,544	122,729,738	22,194

### Analysis of P2X4 and P2X7 expression levels

To test the possibility of P2X4 overexpression, quantitative RT-PCR and Western blot analysis were performed on brain and lung tissue, respectively, of both mouse models. As expected and shown in Fig. 1B,C, both P2X4 RNA and protein levels were about ten and eightfold higher, respectively, in the sEGFP mouse model. Surprisingly, also P2X7 RNA and protein levels were about threefold higher in this mouse. In contrast, both P2X4 and endogenous P2X7 protein levels were unaltered in the P2X7-EGFP mouse (see also^24^). Next, we performed immunofluorescence staining with a P2X4-specific antibody to investigate if the BAC transgenic P2X4 expression in the sEGFP model mirrors the endogenous P2X4 expression in wild type (wt) mice (Fig. 1D). In agreement with the above observations, a clearly stronger P2X4 signal is seen in cerebellar brain slices from the sEGFP reporter model, although this appears less evident in the hippocampus. The P2X4 expression pattern mirrors that of the endogenous P2X4 transcripts (^27,28^, Allen brain atlas) as well as the fluorescent protein patterns described in a BAC transgenic P2X4 reporter mouse line that expresses soluble tdTomato and a recently described conditional P2X4-mCherry knock-in mouse model^18,29^. In these studies, P2X4 transcripts or immunofluorescence were generally seen in Purkinje cells as well as the pyramidal and granular cell layers of the hippocampus and in cortical neurons. As shown in Supplementary Fig. 1, P2X4 staining in Purkinje cells of the sEGFP mice was even strong enough to reveal a pattern that supports its intracellular localisation^30–32^.

**Figure 2:**
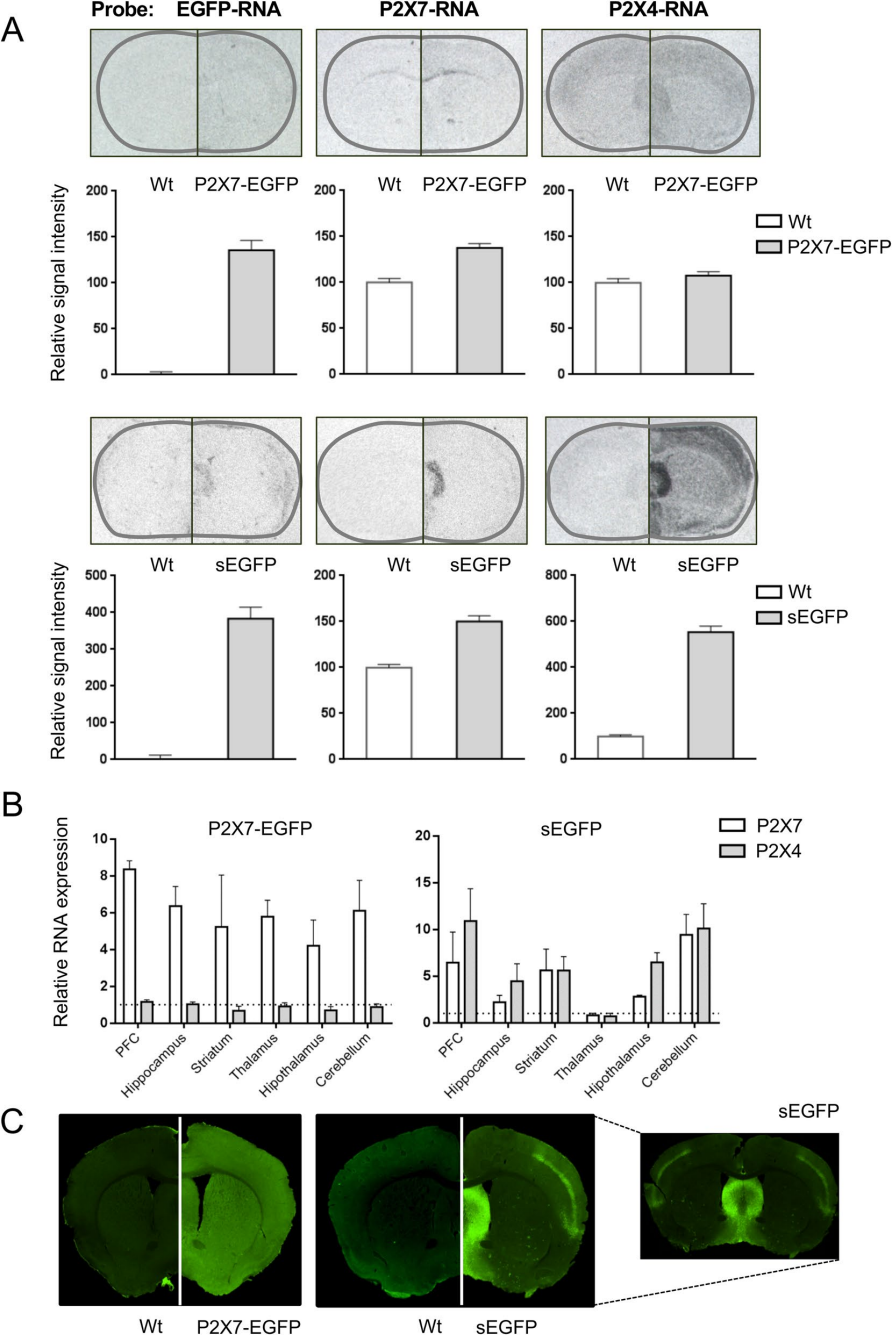
Comparison of EGFP, P2X7, and P2X4 transcript levels and expression patterns in sEGFP and P2X7-EGFP BAC transgenic P2X7 reporter mice. (**A**) In situ hybridization analysis of EGFP, P2X7, and P2X4 transcript levels in the two reporter mouse models in comparison to wt animals. Representative coronal sections (Bregma 0.62 approximately) are displayed. The signal intensities of 12 representative areas were measured on three different mice per group. The background noise was subtracted and the resulting values were normalized to values in wt animals. Bars represent mean ± SEM from three animals analysed in three independent experiments. (**B**) Region-specific quantitative PCR of P2X7 and P2X4 mRNA levels in the P2X7-EGFP and sEGFP mouse lines. RNA levels were normalized to levels in wt animals as well as a housekeeping gene (HPRT) which correspond to the dotted line. Bars represent mean ± SEM from three animals. (**C**) Immunofluorescence staining for EGFP in the P2X7-EGFP and sEGFP lines. Coronal sections from approximately Bregma 0.62 are shown.

Regarding the overexpression of P2X7, we wondered if this could be accounted for by the expression of the P2X7k variant^33^. This functional variant has the same length as the canonical P2X7a variant and is derived from the use of an alternative exon 1´ (compare Fig. 1A) that is present in the first intron of the *P2rx7* gene. To determine if the increased P2X7 transcript and protein levels in the sEGFP model represents P2X7a, maybe as a consequence of some "leakiness" of the introduced poly(A) signal, or if the additional P2X7 protein represents P2X7k that likely has its own regulatory sequences within intron 1, we also performed quantitative RT-PCR with specific primer pairs for both variants. However, in agreement with a lower expression of this variant in brain tissue^33^ significant P2X7k levels could only be detected if PCR cycle numbers were increased. Under these conditions, the relative increase of transcript levels of the k and a variant were similar in the sEGFP (about twofold) and P2X7-EGFP mice (about sevenfold). This suggests that both variants underlie similar regulation mechanisms in both mice but that the k variant is expressed at much lower levels (Supplementary Fig. 2).

### Expression pattern of sEGFP and P2X7-EGFP transcripts in the brain

To compare the expression pattern and region-specific expression levels of EGFP, *P2rx7*, and *P2rx4* transcripts in the two transgenic lines, we next carried out in situ hybridization (ISH) and quantitative RT-PCR in different brain areas (Fig. 2A). ISH revealed a clear EGFP signal and, in agreement with the quantitative PCR data described above, increased *P2rx7* mRNA levels throughout the brain of both mouse models. No differential pattern of P2X7 or EGFP expression was observed between wt and P2X7-EGFP overexpressing mice. In contrast, both the EGFP and P2X7 expression patterns in the sEGFP mouse line showed a clear signal in a region corresponding to the lateral septum, which was not seen in the wt mice. Similarly, a strong overexpression of *P2rx4* transcripts was detected in this same area as well as throughout the brain, which is not observed in P2X7-EGFP mice. A more detailed analysis of *P2rx7* and *P2rx4* expression levels in different brain regions by region-specific quantitative RT-PCR revealed a relatively even distribution of *P2rx4* and *P2rx7* transcripts in all investigated brain regions of the P2X7-EGFP mouse while both *P2rx4* and *P2rx7* levels in the sEGFP mouse varied considerably between regions (*P2rx7*: two to tenfold, *P2rx4*: five to 11-fold) with lowest expression of both transcripts in the thalamus and highest expression in the prefrontal cortex and cerebellum (Fig. 2B).

### Expression pattern of sEGFP and P2X7-EGFP protein in the brain

To understand whether the *P2rx7* and EGFP expression patterns observed by ISH is translated into protein expression, we first performed immunofluorescence stainings with an anti-GFP antibody (Fig. 2C). While the EGFP staining pattern was homogenous in P2X7-EGFP transgenic mice, staining in slices of the sEGFP mouse showed again a clear signal in the lateral septum, as well as in cortical layers confirming the mRNA expression pattern observed by ISH (Fig. 2A). The same pattern was observed in both hemispheres and in several mice, indicating that it is region-dependent and does not represent an irregular expression.

**Figure 3:**
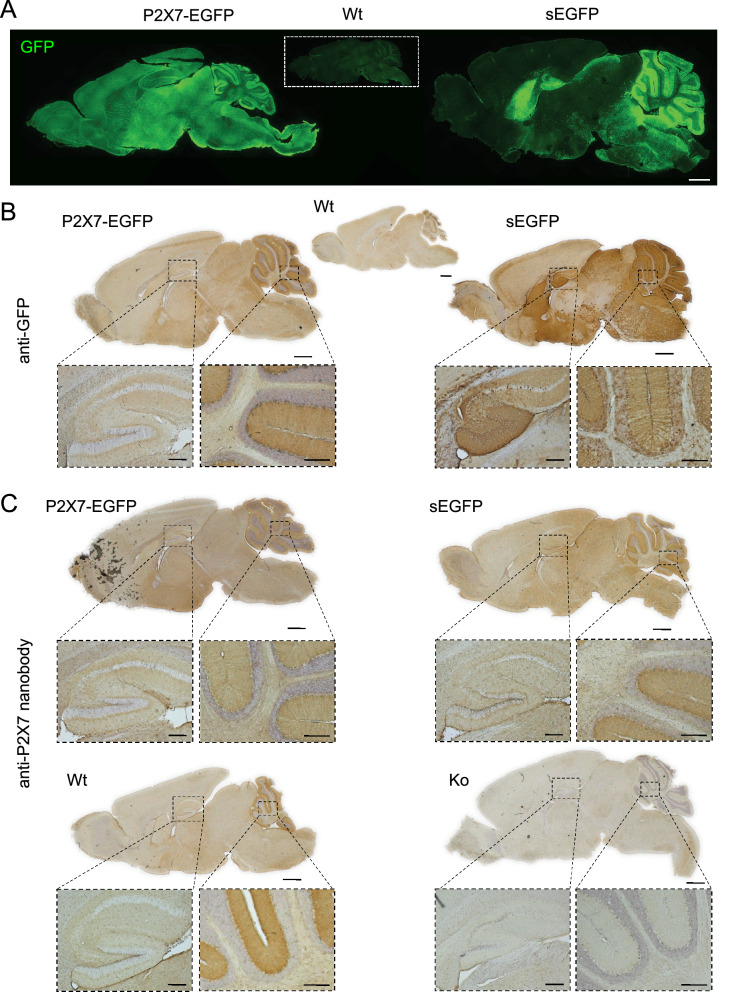
Comparison of EGFP and P2X7 protein expression patterns in sagittal brain sections of sEGFP and P2X7-EGFP BAC transgenic P2X7 reporter mice. (**A**) Immunofluorescence staining with an anti-GFP antibody. Note that signal intensities were individually adjusted to prevent saturation in the sEGFP mouse. Scale bars 1 mm. (**B**) DAB staining with an anti-GFP antibody. (**C**) DAB staining using a P2X7-specific nanobody. Scale bars: 1 mm in whole brain, 200 µm in insets. Representative stainings from 3 different animals per group are shown.

To further compare the overall expression pattern of both reporter constructs, we next performed immunofluorescence stainings on sagittal brain sections. As seen in Fig. 3A, both reporter mouse models show a dominant EGFP staining in the molecular layer of the cerebellum. However, both the distribution and expression levels of EGFP clearly differed in the cerebrum. As shown before^24^, P2X7-EGFP displays a relatively even distribution in all brain regions with more dominant staining in the cerebral cortex, olfactory bulb, thalamus, hypothalamus, substantia nigra, ventral pons, and a fine rim in the molecular layer that is adjacent to the granular layer of the dentate gyrus. In contrast, the soluble EGFP shows a much more localised expression in areas such as the caudate putamen, the superior colliculus and the granular and entire molecular layer of the dentate gyrus, while staining was very weak or even absent in the olfactory bulb and sparse in the pons. A diffuse expression is present in the hypothalamus.

To further confirm this and to allow comparison with the endogenous P2X7 distribution in wt mice, we next performed DAB stainings with an anti-GFP antibody (Fig. 3B) and a P2X7-specific nanobody (Fig. 3C). As seen in Fig. 3B, DAB staining of EGFP in the two reporter mouse models confirmed the overall expression pattern obtained by immunofluorescence staining and revealed further differences between both models. While P2X7-EGFP staining was enhanced in an approximately 50 μm wide rim surrounding the granular layer of the dentate gyrus and showed a more or less uniform distribution in the other hippocampal areas (as previously described^24^), sEGFP staining was dominant throughout the entire molecular layer of the dentate gyrus and, in addition, in the granular cell layer. In addition, a strong EGFP signal was found in large single cells with neuron-like morphology across the pyramidal cell layer of the CA region. In the cerebellum, a similar staining pattern of the molecular layer was obtained in both mouse models. However, again a clear staining of single cells within the granular layer was found in the sEGFP model and absent in the P2X7-EGFP mouse.

Since the colorimetric DAB staining gave a more intense signal with the P2X7-specific nanobody than immunofluorescence staining, this method also allowed comparison of the P2X7 expression in the reporter mouse models with that of endogenous P2X7 in wt animals. As seen in Fig. 3C, both BAC transgenic mouse models mirror the expression pattern seen in wt mice, although, as expected, with a higher intensity due to the P2X7 overexpression in both models. Specificity of the P2X7 staining was confirmed using P2X7 knockout mice. Importantly, the EGFP and P2X7 expression patterns were markedly different in the sEGFP mouse.

### Cell type-specific expression of EGFP in the two reporter mouse models

**Figure 4:**
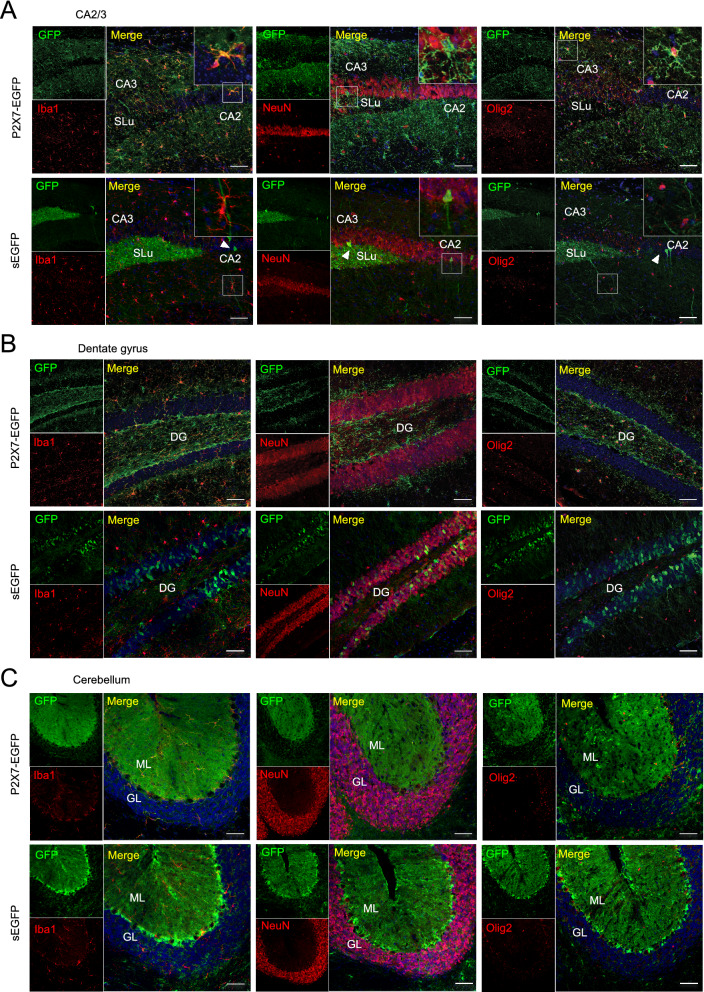
Cell type-specific EGFP protein expression in in sEGFP and P2X7-EGFP BAC transgenic P2X7 reporter mice. Brain slices from BAC transgenic mice were co-labelled with an anti-GFP antibody and antibodies against marker proteins for microglia (Iba1), neurons (NeuN), and oligodendrocytes (Olig2). Representative results are shown for the CA3-CA2 border (**A**), the dentate gyrus (**B**) and cerebellum (**C**). DAPI was used as nuclear counter stain (blue). Arrowheads indicate cells with neuron-like morphology in the CA regions. SLu, stratum lucidum; CA, cornu ammonis; DG, dentate gyrus; ML, molecular layer; GL, granular layer. Scale bar: 50 μm.

**Figure 5:**
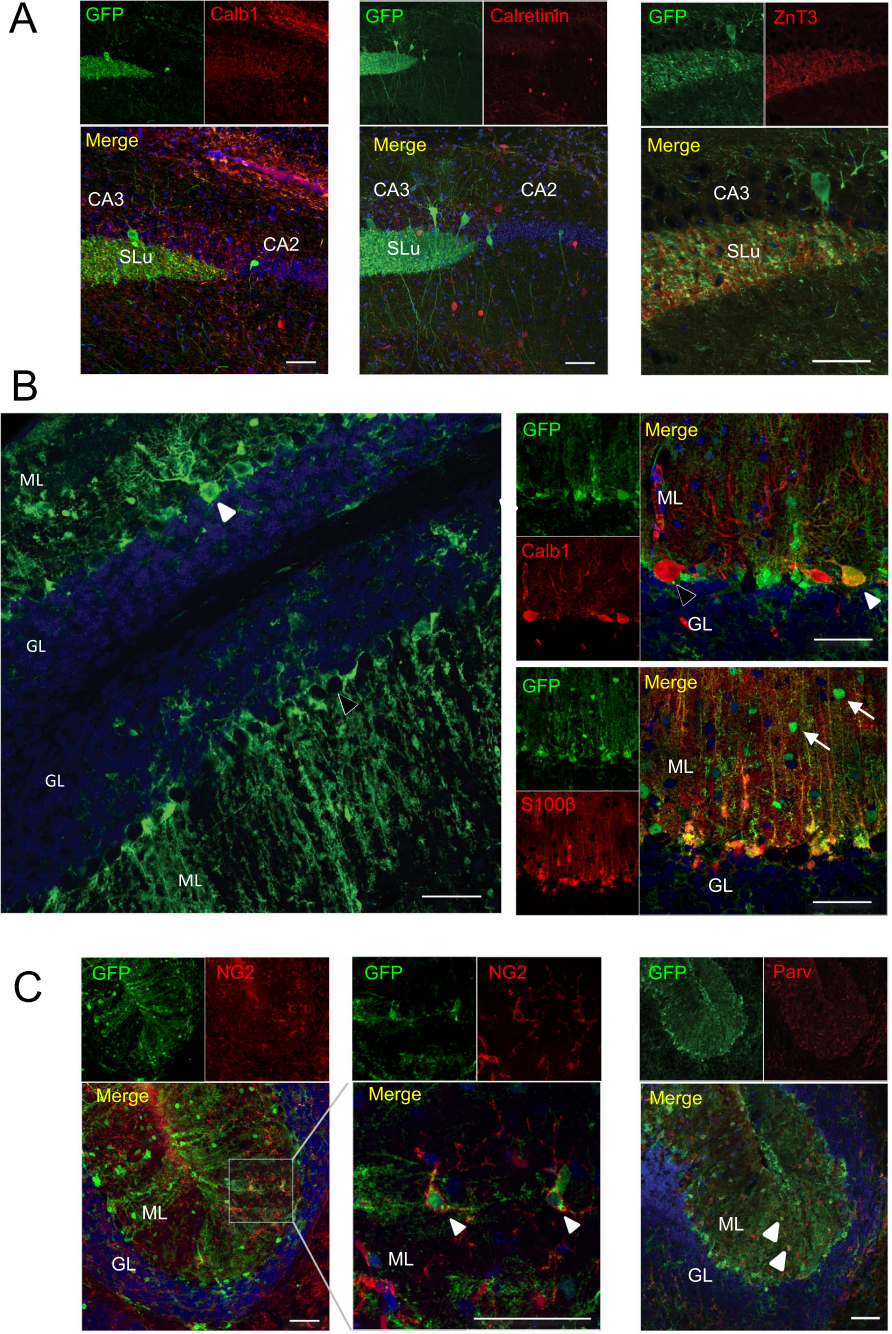
sEGFP-positive cells in the hippocampus and sporadic sEGFP expression in Purkinje cells. Brain slices from BAC transgenic sEGFP mice were co-labelled with an anti-GFP antibody and antibodies against marker proteins for neuronal and glial cell types. Representative immunofluorescence stainings are shown. (**A**) Immunofluorescence staining in the CA3-CA2 border with antibodies against calbindin-1 and calretinin (GABAergic interneurons) and in the CA3 region with an antibody against ZnT3 (synaptic vesicles). (**B**) Staining in the cerebellum with antibodies against Calbindin-1 (Purkinje cell) and S100β (Bergman Glia**).** Single EGFP-positive cells show Purkinje cell morphology (white arrowheads). Note that not all Purkinje cells are EGFP-positive (black arrowhead). White arrows indicate an unidentified cell type in the molecular layer. (**C**) Staining in the cerebellum with antibodies against NG2 (oligodendrocyte progenitor cells) and parvalbumin (Purkinje cells and molecular layer interneurons). Examples of double-positive cells are pointed out using white arrowheads in each case. DAPI staining is shown in blue. CA, cornu ammonis; ML, molecular layer; GR; granular layer. Scale bar: 50 μm.

To identify the respective EGFP-expressing cell types, we next performed double immunofluorescence stainings of EGFP together with cell type-specific marker proteins in the dentate gyrus, at the CA3-CA2 border, and in the cerebellum (Fig. 4). In agreement with previous findings, P2X7-EGFP was co-localised in all regions with the microglial marker protein Iba1 and the oligodendrocyte marker protein Olig2 but was absent in NeuN-positive neurons. In contrast, soluble EGFP was not detected in microglia and oligodendrocytes but co-localised with 16.33 ± 5.29% of the NeuN-positive neurons in the granular layer of the dentate gyrus (Fig. 4B) and sporadically with cells showing neuron-like morphology in the CA regions (arrowheads in Fig. 4A). The CA3-CA2 border was chosen because the sEGFP mouse revealed also a strong EGFP staining in the stratum lucidum, the suprapyramidal tract of unmyelinated inhibitory and excitatory mossy fibre projections. The EGFP signal in this region might originate from projections of the EGFP-positive granule cells in the DG. No clear co-localisation of soluble EGFP with the synaptic marker proteins calretinin or calbindin-1 (Calbindin D28k) for GABAergic interneurons or the vesicular Zinc transporter 3 (ZnT3) was found in this region (Fig. 5A).

In the cerebellum, both mice showed intense EGFP staining of Bergmann glia in the molecular layer (Figs. 4C, 5B, compare also^24^). In addition, sEGFP but not P2X7-EGFP^24^ was sporadically (< 10% of the cells) found in Purkinje cells (white arrowheads in Fig. 5B) and an unidentified cell type, likely stellate cells or basket cells, in the molecular layer (arrows in Fig. 5B). This assumption is supported by the partial co-localisation of EGFP with the protein marker parvalbumin that has been shown to stain both cell types (Fig. 5C). Furthermore, some of the EGFP-positive cells in the molecular layer were NG2-positve (Fig. 5C), but the overlap was only sporadic and the majority of NG2-positive cells was EGFP-negative.

Finally, there was no co-localisation between the astroglial marker GFAP and EGFP in either mouse model (Supplementary Fig. 3). Thus, cell type-specific EGFP expression appears to be almost opposed in both reporter mouse models with at least partial neuronal expression in sEGFP mouse, and dominant microglial and oligodendrocytic expression in the P2X7-EGFP mouse.

### Analysis of EGFP expression in immune cells in the two reporter mouse models

**Figure 6:**
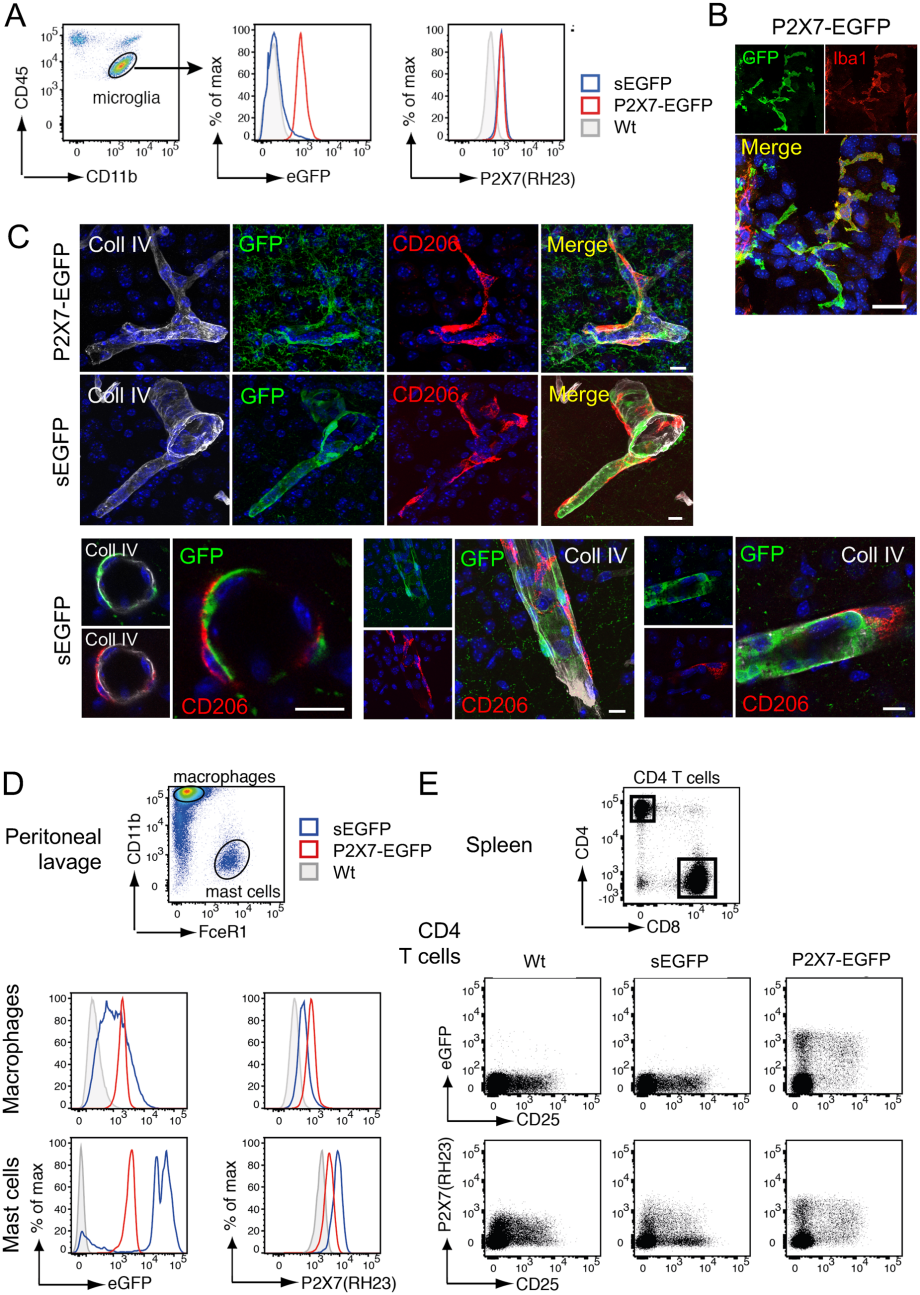
Comparison of EGFP expression in immune cells of the brain. (**A**) Brain microglia (CD11^b^CD45^low^) from wt and BAC transgenic mice were analysed by flow cytometry for endogenous EGFP expression and cell surface P2X7 expression using a monoclonal antibody (clone RH23A44). (**B**) Immunofluorescence staining of Iba1 and EGFP in the third ventricle of the P2X7-EGFP mouse. Scale bar: 25 μm. (**C**) Comparison of perivascular macrophage staining in sEGFP and P2X7-EGFP mice. Anti-collagen IV antibody was used to stain blood vessels and CD206 was used as a marker for perivascular macrophages. Lower panel: detailed images from the sEGFP mouse showing a lack of overlap between EGFP and CD206 staining. Scale bar:10 µm, DAPI staining in blue. (**D**) Peritoneal macrophages (CD11b^+^FceR1^–)^ and mast cells (CD11b^–^FceR1^+^) from wt and BAC transgenic mice were analysed by flow cytometry for endogenous EGFP expression and cell surface P2X7 expression. (**E**) CD4^+^ T cells from the spleen wt and BAC transgenic mice were analysed by flow cytometry for endogenous EGFP expression and cell surface P2X7 expression. EGFP and P2X7 expression levels were compared on helper T cells (CD4^+^CD25^–^) and regulatory T cells (CD4^+^CD25^+^).

To confirm the unexpected absence of EGFP in microglia of the sEGFP model, we next performed FACS analysis of microglia. As seen in Fig. 6A, staining with an anti-P2X7 antibody revealed increased P2X7 levels in the two reporter mouse models, in agreement with the increased P2X7 levels observed in the sEGFP mouse and the expected P2X7-EGFP overexpression, respectively. Detection of the direct fluorescence of the EGFP reporter in flow cytometric analyses, however, indicated an almost complete absence of EGFP fluorescence in microglia from the sEGFP mouse, which was comparable to that of wt microglia. In contrast, the histogram of microglia from P2X7-EGFP mice showed a clear right shift, as expected for EGFP-expressing cells.

Since P2X7 is best known for its function in macrophages and co-immunostaining in the third ventricle of P2X7-EGFP mouse (Fig. 6B) suggested its presence in Iba1-positive cells with a macrophage-like morphology, we next analysed its presence in macrophages of the brain. Using an antibody against the plasma membrane mannose receptor CD206, a marker protein for perivascular macrophages, confirmed the presence of P2X7-EGFP in this cell type but no other vessel-associated cells (Fig. 6C). The only other cells that were stained had microglia-like morphology. In the sEGFP model, however, no co-staining of EGFP with CD206-positive cells was observed. Instead, EGFP staining was identified in two other cell types on the outer side of the collagen-stained basement membrane of the vessel. Based on their morphology, these cells most likely corresponded to pericytes (extended, spindle-shaped) and smooth muscle cells (surrounding the vessel with split edges).

These unexpected findings prompted us to also compare EGFP reporter expression in peripheral immune cells. In peritoneal macrophages and mast cells of wt mice, a uniform population of endogenous P2X7 was clearly detected by staining with an anti-P2X7 antibody and the right-shifted histograms confirmed again its overexpression in both reporter mouse models (Fig. 6D). A similar shift and shape of the histogram was obtained when cells of the P2X7-EGFP mouse were analysed for EGFP fluorescence, confirming the overlap between (endogenous) P2X7 and EGFP expression in these mice. In contrast, clearly different histograms were seen in sEGFP mice. In macrophages, a very broad histogram was obtained indicating a high variability in EGFP expression levels ranging from macrophages with very low EGFP expression (overlap with the EGFP signal in wt mice) to macrophages with an EGFP signal that was even higher than in P2X7-EGFP overexpressing mice. In mast cells, at least three populations could be differentiated: a small population that showed no EGFP expression at all and two populations with exceptionally high EGFP expression, which was higher than that observed in P2X7-EGFP mice. Finally, analysis of spleen-derived CD4^+^ T cells (Fig. 6E) revealed an almost complete absence of EGFP in regulatory T cells/activated helper T cells (CD4^+^CD25^+^) and helper T cells (CD4^+^CD25^–^) from sEGFP mice, although cell surface P2X7 expression could be verified by using the anti-P2X7 antibody. In contrast, P2X7-EGFP CD4^+^ T cells showed a similar staining pattern when comparing EGFP and cell surface P2X7 staining. In summary, the sEGFP mice show absence of the EGFP reporter in microglia and CD4^+^ T cells, highly variable expression in macrophages and mast cells, with very strong expression in some mast cell populations. Overall, this pattern is strikingly different to that of the endogenous P2X7 receptor in wt mice, which is well reproduced in the P2X7-EGFP model.

### Detection of a functional phenotype in the sEGFP reporter mouse model

**Figure 7:**
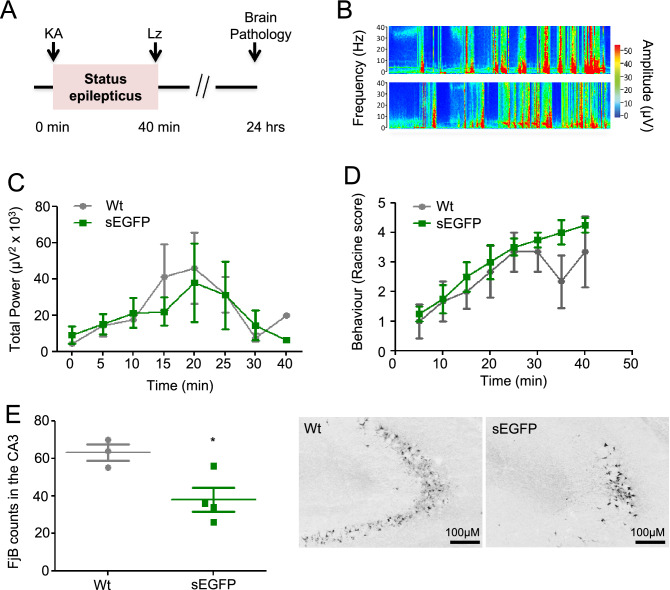
Functional phenotype of the sEGFP reporter mouse. (**A**) Experimental design of the intra-amygdala mouse model of status epilepticus. Status epilepticus is induced by an injection of 0.2 µg kainic acid (KA) into the basolateral nucleus of the ipsilateral amygdala. To reduce mortality and morbidity, the anti-convulsant lorazepam (Lz) is administered 40 min following KA injection. (**B**) Representative EEG recordings presented as heat maps of frequency and amplitude data during status epilepticus of wt and sEGFP mice. (**C**) Graphs showing similar seizure severity between wt and sEGFP mice during a 40 min recording period starting at the time of intraamygdala KA injection until administration of anticonvulsant lorazepam (wt, n = 3 and sEGFP, n = 4). (**D**) Graph showing similar changes in behaviour based on the Racine scale between genotypes (wt, n = 3 and sEGFP, n = 4). (**E**) Graph and photomicrographs (10 × lens) showing less FjB-positive cells in the ipsilateral CA3 subfield of the hippocampus 24 h after status epilepticus in sEGFP mice compared with wt mice (n = 3 (wt), 63.00 ± 4.359; n = 4 (sEGFP), 38.00 ± 6.377; t = 2.983 df = 5, unpaired t-test; p = 0.031). Scale bar, 100 μm.

Both P2X7 and P2X4 have previously been shown to be involved in seizure induction and seizure-induced cell death^20,34,35^. Therefore, we undertook a preliminary study to test whether the overexpression of P2X4 and/or P2X7 in the sEGFP mouse has functional consequences in these processes. sEGFP mice and wt littermates were subjected to *status epilepticus*^20^ (Fig. 7A) via a microinjection of KA into the basolateral amygdala^36^. No difference was found in baseline EEG recordings between wt and sEGFP mice (total power: 4431 ± 814.2 μV^2^ wt vs. 5130 ± 948.4 μV^2^ sEGFP, *p* = 0.6172, n = 3 wt and 4 sEGFP) and both wt and sEGFP mice experienced similar seizure severity during a 40 min recording period starting at the time-point of KA injection until the administration of the anticonvulsant lorazepam (total power: 32,350 ± 10,830 µV^2^ wt vs 23,280 ± 10,470 µV^2^ sEGFP; *p * = 0.581, n = 3 wt and 4 sEGFP, Fig. 7B,C). Likewise, wt and sEGFP mice displayed similar behaviour changes during status epilepticus (Fig. 7D) and additional EEG recordings for 60 min post-lorazepam administration showed no difference between genotypes (25,990 ± 11,610 µV^2^ wt vs 20,320 ± 7196 µV^2^; *p * = 0.679, n = 3 wt and 4 sEGFP). As intraamygdala KA-induced *status epilepticus* leads to characteristic lesions within the ipsilateral CA3 subfield comprising loss of selected neurons and gliosis, we also analysed hippocampal brain sections to compare the neuropathological outcomes. Here, sEGFP mice showed less neurodegeneration in the CA3 subfield of the ipsilateral hippocampus as evidenced by fewer FjB-positive cells (Fig. 7E). Thus, although showing no obvious differences in seizure threshold to intraamygdala KA-induced status epilepticus, neurodegeneration post-status epilepticus was reduced in sEGFP mice, suggesting that the increased expression of P2X4 and P2X7 has an impact on pathological processes.

## Discussion

In this study, we analysed the P2X4 and P2X7 expression levels and the EGFP expression patterns in two BAC transgenic P2X7 reporter mouse models and compared it with the endogenous P2X7 distribution in wt mice. Our data show overexpression of a *P2rx4* passenger gene, and surprisingly also of the *P2rx7* gene, in the sEGFP model. We further demonstrate that the expression pattern of soluble EGFP can be reconciled with partial neuronal expression while the P2X7-EGFP fusion protein is dominantly expressed in microglia and oligodendrocytes but not detectable in neurons. Most surprisingly, sEGFP expression appeared to be largely absent in microglia and macrophages, at least under physiological conditions. It has to be noted though, that sEGFP expression was previously observed in microglia in models of *status epilepticus*^37^ and Alzheimer´s disease^23^, suggesting that it becomes upregulated under pathophysiological conditions. We conclude that the sEGFP reporter shows a partially aberrant expression pattern and preliminary functional data suggest that the observed P2X4 and/or P2X7 overexpression in the sEGFP mouse could influence outcomes during pathology.

BAC transgenic reporter mouse models have revolutionized the analysis of protein expression in vivo*.* They are widely used to study general or cell type-specific protein expression and to isolate and analyse specific target cells or proteins^14^. This methodology has been particularly useful to study complex cell systems such as the CNS^17,38^ and more than 1000 BAC transgenic reporter, Cre-driver, and TRAP mouse lines have been generated and initially characterized within the GENSAT project^39^, representing an invaluable resource for researchers. Considering the efficiency and large throughput with which these lines are generated^19^, it is evident, that they can only be preliminary characterized and further in-depth analysis is required. In particular, limitations that are inherent to the BAC transgenic approach need to be considered, such as variability in the integrated BAC copy number, non-specific integration of the BAC in the genome and alterations in the gene structure and regulation of both the target gene on the BAC and genes that may be affected by the BAC integration. The need for careful validation of reporter mouse lines was shown, for example, in a comparative analysis of three transgenic mouse models for neuronal expression of the corticotropin-releasing hormone (CRH). Here, differences appeared to depend not only on the transgenic approach (IRES-CRE, BAC or Cre-loxP system targeting the *Crh* gene promoter) but also on the reporter used (GFP or tdTomato)^40^. In another example, leakiness of Cre-dependent reporter lines (resulting in unspecific EGFP distribution) and lack of specificity of BAC-derived Cre-driver lines for several microglia-specific Cx3cr1-Cre lines have recently been reported^41^. While knock-in approaches provide a more controlled way of gene modification^42^, the BAC transgenic approach has the advantage of being fast and highly efficient. Furthermore, it allows the moderate overexpression of the target gene, which might help its efficient visualization and the analysis of overexpression effects.

While the P2X4 overexpression in the sEGFP mouse model is inherent to the BAC clone chosen for modification, the reason for the increased P2X7 expression level and divergent expression pattern in this model is not immediately clear. In the sEGFP construct, the *P2rx7* gene is practically interrupted by the EGFP encoding sequence followed by a pA signal and the targeting vector while in the P2X7-EGFP mouse, the *P2rx7* gene structure was largely unaltered, except for the EGFP sequence insertion. The pA signal following the sEGFP insertion should prevent formation of BAC-derived *P2rx7* transcripts in this model. Thus, sEGFP can be regarded as a read-out of *P2rx7* promoter activation while the P2X7-EGFP reporter mouse leads to the overexpression of the translated EGFP-tagged *P2rx7* gene product. Regarding the observed P2X7 overexpression, sequencing of the integration sites of the EGFP cDNA revealed that the EGFP-encoding sequence plus an upstream 27 bp and the downstream targeting vector sequence were inserted directly after the A of the P2X7 start ATG (Supplementary Fig. 4). Due to the used recombination strategy, in which the BAC was not resolved^19^, this resulted in duplication of the homology domain (ranging from -332 bp upstream of the transcription start site right to the start ATG (+ 163 bp downstream of the transcription start) and complementation of a second P2X7 start ATG at the 3´ integration site. According to a previous study^21^, a fragment ranging from − 249 bp to + 220 bp was already sufficient for reporter gene expression and can therefore be assumed to contain essential promoter elements. Nevertheless, since the P2X7 overexpression is much lower than that of the P2X4 receptor, its expression might be partly prevented by the introduced pA signal. Finally, it cannot be excluded, that the P2X4 overexpression affects the P2X7 expression as mutual interactions between them have been described^43^.

Regarding the different expression pattern in the sEGFP mouse, one explanation could be that the accessibility or structure of domains required for the regulation of P2X7 expression is affected by the described gene modification. Also, the posttranscriptional regulation of the sEGFP is likely different from that of the P2X7-EGFP as its expression should result in the generation of the much shorter EGFP transcript in which intronic *P2rx7* sequences and the 5`UTR are missing. For example, the first intron, which is not preserved in the sEGFP transcript, is particularly long in many genes and supposed to contain elements that are important for gene regulation. Intron 1 of *P2rx7*, for example, might contain elements that control expression of the P2X7k splice variant^33^. In addition, sequences encoding or binding regulatory RNAs might be eliminated and also other post-transcriptional mechanisms could affect protein expression such as RNA editing^44^, changes in poly(A) tail length^45^, mRNA capping, mRNA splicing, or protein turnover. For example, P2X7 has been shown to be targeted by several microRNAs, such as mir-22^46^, and it cannot be excluded that this suppresses the expression of P2X7 or its EGFP-tagged version in neurons. Likewise, the strongly reduced or absent sEGFP expression in microglia, perivascular macrophages of the brain, and T cells indicates a lack of positive regulatory mechanisms.

It further needs to be considered that cytoplasmic sEGFP and the membrane bound P2X7-EGFP fusion construct have different cellular distribution. In cells like neurons with long and fine processes, this can result in apparent differences in the EGFP reporter localisation (e.g. in regions of the cell body like granular layers or the projection areas such as the mossy fiber area, Fig. 4A,B) and very different appearance of the respective staining (e.g. punctate and diffuse for membrane localised P2X7-EGFP as in the cerebellar Bergmann glia or filling the whole cell and representing the respective cell morphology as in the case of sEGFP in these cells, Fig. 4C). Thus, neuronal expression of membrane bound P2X7-EGFP might be difficult to detect in the complex cellular context of a brain slice if the protein is localised to discrete and tiny structures such as specific synapses or growth cones. In addition, the sEGFP mouse shows sparse, diffuse EGFP staining of cells with an irregular and variable shape throughout different brain areas (i.e. pons, superior colliculus, caudate putamen, compare Fig. 3). This is very similar to the staining described in the GENSAT database, which has been attributed to glial cells (www.gensat.org). However, this could not be confirmed using the glial markers Iba1, Olig2, and GFAP (data not shown). Likewise, no clear co-localisation with the neuronal marker NeuN was found in these regions, neither in sagittal nor coronal sections.

While BAC transgenic technology has greatly advanced neuroscience research, several caveats need to be considered and reporter lines need to be carefully planned and thoroughly examined. P2X7 reporter mice provide important tools for the elucidation of P2X7 receptor roles in CNS diseases. However, the presence of passenger genes and leaky or aberrant reporter expression may confound data interpretation and needs to be considered. Our data raise concerns about the reliability of the sEGFP mouse model and suggest that conclusions drawn from previous localisation studies need to be carefully re-evaluated.

## Material and methods

### Animals

Tg(P2rx7 EGFP)FY174Gsat (sEGFP) mice were generated by the Gene Expression Nervous System Atlas (GENSAT) project (https://www.gensat.org) and obtained from the Mutant Mouse Resource and Research Center (MMRRC). They were bred in FVB/N background or C57BL6 (only Fig. 2). P2rx7^tmid(EUCOMM)Wtsi^ were in C57BL/6 background. Tg(RP24-114E20P2X7451P-StrepHis-EGFP)17Ani (P2X7-EGFP, line 17) mice^24^ were in FVB/N (Fig. 1B) or C57BL/6 N (all other data) background. No differences in P2X7-EGFP or P2X7 expression were identified between both backgrounds (compare respective stainings from C57Bl/6 N (in this study) and FVB/N^24^ and see supplementary Fig. 5). Male and female mice were used in all experiments. Mice were housed in standard conditions (22 °C, 12 h light–dark cycle, water/food ad libitum). All animal experiments were performed in accordance with the principles of the European Communities Council Directive (2010/63/EU). Procedures were reviewed and approved by the Research Ethics Committee of the Royal College of Surgeons in Ireland (REC 1322) and from the Health Products Regulatory Authority (HPRA, AE19127/P038) and the State of Upper Bavaria (55.2–1-54–2532-59–2016). All efforts were made to minimize suffering and number of animals.

### Sequencings of sEGFP insertion site

The genotyping primers recommended for the sEGFP mouse (F: sequences Exon 1 of *P2rx7,* upstream of the Start ATG 5′-CGCTGCAGTCACTGGAGGAA-3′ R: EGFP 5′-TAGCGGCTGAAGCACTGCA-3′) and a primer pair consisting of a forward primer in the EGFP sequence (5′-TGCAGTGCTTCAGCCGCTA-3′) and a reverse primer in the coding sequence of exon 1 (5′-GTGGGTCTTGCACATGATCGTCT-3′) were used to amplify a 399 bp and a 3831 bp fragment, respectively from mouse tail DNA. From these fragments, the targeting vector sequence and the 5´ and 3´insertion sites were determined by sequencing (Eurofins, Munich, Germany).

### RNA extraction and real-time PCR

RNA was extracted from one hippocampus using the QIAzol Lysis Reagent (Qiagen, Hilden, Germany) as described^47^ and 500 ng of total RNA were reverse transcribed with SuperScript II (Thermo-Fisher, MA, USA). Quantitative real-time PCR was performed using the QuantiTect SYBR Green PCR Kit (Qiagen, Hilden, Germany) and 1.25 µM of each primer pair in combination with a LightCycler 1.5 (Roche Diagnostics GmbH, Mannheim, Germany) according to the manufacturer’s protocol. Data were analysed with the LightCycler 1.5 software and normalized to expression of β-actin. Primers were designed using Primer3 software (https://frodo.wi.mit.edu) and had the following sequences: *P2rx7* forward (F) 5′-ACTGGCAGGTGTGTGTTCCATA-3′ and reverse (R): 5′-TTGGCAAGATGTTTCTCGTG-3′; *P2rx4* F: 5′-TATGTGGTCCCAGCTCAGGA-3′ R: 5′-TCACAGACGCGTTGAATGGA-3′ EGFP F: 5′-ACGTAAACGGCCACAACTTC-3′ R: 5′-AAGTCGTGCTGCTTCATGTG-3′. To detect and differentiate alternatively spliced *P2rx7* transcripts we combined exon 1 and exon 1′ forward sequences (5′-CACATGATCGTCTTTTCCTAC-3′ and 5′-GCCCGTGAGCCACTTATGC-3, respectively) with reverse primers in exon 4 (5′-GGTCAGAAGAGCACTGTGC-3′), exon 5 (5´-CCTTGTCTTGTCATATGGAAC-3′), and exon 7 (5′-TCTGTAAAGTTCTCTCCTGC-3′) and increased the number of amplification cycles from 40 to 55.

*Tissue-specific real-time PCR* Tissue punches of different brain areas were collected from frozen samples. Total RNA was extracted using the miRNeasy Mini Kit (QIAGEN). First-strand cDNA was produced by reverse transcription employing the Superscript III Reverse Transcriptase kit (ThermoFisher Scientific) with Oligo(dT)12–18 Primer (ThermoFisher Scientific). Relative levels of gene expression were obtained through quantitative real-time PCR carried out in a LightCycler 96 instrument (Roche) following the FastStart Essential DNA Green Master kit (Roche). The primer sequences employed for the testing were as follows: *P2rx7* F: 5-ACTGGCAGGTGTGTTCCATA-3′. R: 5′-TTGGCAAGATGTTTCTCGTG-3′, *P2rx4* F: 5′-TATGTGGTCCCAGCTCAGGA-3′, R: 5′-TCACAGACGCGTTGAATGGA-3′, HPRT (housekeeping) F: 5′-TGGGCTTACCTCACTGCTTTCC-3′, R: 5′-CCTGGTTCATCA TCGCTAATCACG-3′. Results were normalized to HPRT, as well as to wt animal values.

### SDS-PAGE and Western Blot analysis

P2X protein was analysed as described^24^. 100–500 mg of lung tissue were cut into small pieces and homogenized in 600 µl of homogenization buffer (0.1 M sodium phosphate buffer, pH 8.0, 0.4 mM Pefabloc SC (Sigma) and Complete protease inhibitor (Roche Applied Science) using a Precellys homogenizer (Peqlab) and CK28 beads (15 s, 5.000 rpm). After centrifugation (10 min at 10,000 × *g*, 4 °C), membranes were pelleted from the supernatant (30–60 min at 21,100×*g*, 4 °C), and extracted for 15 min with 100–300 µl of the above homogenization buffer containing 1% NP-40 (Sigma) as detergent. After centrifugation (10 min at 21,100×*g*, 4 °C) protein concentrations of the supernatants were determined by BCA Protein-Assay (ThermoFisher Scientific) and 30–75 μg of total protein per lane were separated on 8% SDS-PAGE gels using 20 mM DTT (Roth) as reducing agent. Proteins were wet-blotted onto Immobilon-FL PVDF membranes (Merck Millipore) for 16 h at 4 °C and the membranes blocked in Intercept (TBS) Blocking Buffer (LI-COR Biosciences) diluted 1:2 in TBS. Upon immunostaining, proteins were visualized with an Odyssey infrared imaging system (LI-COR Biosciences) and quantified using ImageStudio (LI-COR Biosciences). Statistical analysis was performed using GraphPad Prism.

### In situ hybridization

In situ hybridization was carried out as previously described^48^. Fresh brains were frozen on dry ice and stored at -80 °C. For in situ hybridization, 20 μm cryosections were incubated overnight with gene-specific S^35^-UTP- labelled riboprobes. The nucleotides targeted by the different probes were as follows: EGFP: 907–1698 of GenBank accession no. LC311024; P*2rx4*: 1004–1910 of GenBank accession no. NM_011026; *P2rx7*: 1199–1620 of GenBank accession no. NM_011027. Assessment of the signal intensity was carried out using ImageJ software. Results were normalized to values from wt animals.

### Diaminobenzidine (DAB) immunohistochemistry

Stainings were performed as described^24^. Mice were anesthetized (Ketamine/Xylazine/Heparine) and transcardially perfused with 4% paraformaldehyde (PFA). Brains were dissected and post-fixed in 4% PFA for a minimum of 24 h, transferred to 30% sucrose for cryoprotection for at least 48 h, and embedded in 5% low melting point Agarose (Roth, Germany). 30 µm sagittal brain sections were prepared (VT1200s Leica Microsystems) and, after peroxidase block (1% H_2_O_2_ in PBS, 30 min RT), blocked with 10% Normal Goat Serum (Dako, Germany) and 0.1% Triton X-100 (Sigma, Germany)in PBS (1 h, RT), and incubated with primary antibody overnight at 4 °C. After incubation with biotinylated secondary antibodies (1.5 h at RT), proteins were visualized using the ABC method with the Vectastain ABC kit (Vector Laboratories) and the DAB substrate Sigma Fast DAB Tablets (Sigma-Aldrich, Germany). Brain slices were mounted on glass slides and counter-stained with haematoxylin (Roth), followed by dehydration (70%, 80% and 100% ethanol, xylene) and embedding with phenol-free Kaiser’s glycerol gelatine (Roth). Images were taken with a Zeiss AxioCam MR coupled to a Zeiss Axio Imager M2 using the Zeiss Axiovision software.

### Immunofluorescence staining

Immunostaining was performed as described^49^. Adult mouse brains were fixed and cryoprotected as above and 40 µm cryostat sections were prepared. Tissue slices were washed (3 × times 10 min, PBS), blocked (5% Normal Goat Serum (Dako Germany), 0.3% Triton X-100 (Sigma, Munich, Germany) in PBS, two hr at RT), and incubated with primary antibodies (16–24 h, 4 °C) with gentle shaking. After washing as above, sections were incubated for 2 h with fluorescence conjugated secondary antibodies. DAPI staining was performed for 60–90 s and slices were mounted on object slides with Lab Vision PermaFluor Aqueous Mounting Medium (ThermoFisher Scientific). For perivascular macrophage staining, brains were cut on a vibratome and 50 µm floating sections were collected. Tissue slices were washed (3 times 10 min, TBS), blocked (1% BSA (Sigma, Munich, Germany), 0.5% Triton X-100 (Sigma, Munich, Germany), 0.1% Fish gelatine (Sigma, Munich, Germany) in TBS, 2–3 h at RT), and incubated with primary antibodies (24 h – 5 days, depending on the antibody, 4 °C) with gentle shaking. After washing as above, sections were incubated for 2–3 h with fluorescence conjugated secondary antibodies and DAPI diluted in blocking buffer. Slices were mounted on glass slides with Everbrite Mounting Medium (Biotium, Fremont CA, USA). Images were obtained by confocal laser scanning microscopy (Zeiss LSM 880). Quantitative analysis was performed with the StrataQuest automated cell counting software (TissueGnostics, Vienna, Austria).

### Antibodies for Western blotting and immunohistochemistry

See Supplementary Table 1.

### FACS analysis

#### Isolation of primary cells

Isolation of primary cells was described before^50^. For the isolation of brain microglia, mice were sacrificed under anesthesia by cervical dislocation. Single cell suspensions were prepared from brain by collagenase digestion at 37 °C for 30 min. Cells were passed through a 70 µm cell strainer (Greiner) and centrifuged for 5 min at 300 g. Microglia were separated from debris by percoll gradient centrifugation (33% percoll solution, GE Healthcare). The supernatant was removed and the pellet was resuspended in 1 ml ACK erythrocyte lysis buffer ice for 1 min to remove erythrocytes. Cells were washed with 10 ml FACS buffer (PBS + 0.2% BSA / 1 mM EDTA) and resuspended in FACS buffer.

For the isolation of peritoneal macrophages and mast cells, mice were sacrificed and 5 ml PBS + 1 mM EDTA were injected to lavage the peritoneal cavity. The peritoneal lavage was centrifuged for 5 min at 300 g and the pellet was resuspended in FACS buffer.

For the isolation of spleen T cells, mice were sacrificed, the spleen was collected and processed through a 70 µm cell strainer (Greiner) using a syringe piston. The cell suspension was centrifuged for 5 min at 300 g, erythrocytes were removed by ACK erythrocyte lysis as described above and the cells were resuspended in FACS buffer.

#### Antibodies and flow cytometry

Cells were stained with fluorochrome-conjugated mAbs for 30 min on ice in the presence of Fc block (anti-CD16/CD32; clone 2.4G2, BioXcell) and normal rat serum (Jackson). Staining and washing was performed in FACS buffer containing PBS, 0.1% BSA, and 1 mM EDTA. The following antibodies were used: anti-CD11b (clone M1/70; BioLegend), anti-P2X7 (clone RH23A44, UKE), anti-CD45 (30-F11, Biolegend), anti-CD4 (clone RM4-5; BioLegend), anti-CD8a (clone 53–6.7, Biolegend), anti-CD25 (clone PC61, Biolegend) and anti-FcεR1α (clone MAR1, Biolegend). Cells were analysed using a BD Celesta flow cytometer and data were analysed with FlowJo software (Treestar).

### Intraamygdala kainic acid-induced status epilepticus mouse model

Intraamygdala kainic acid (KA)-induced status epilepticus was performed as described before^20^. Mice were anesthetized using isoflurane (5% induction, 1–2% maintenance) and maintained normothermic by means of a feedback-controlled heat blanket (Harvard Apparatus Ltd, Kent, UK). Under anesthetized conditions, mice were placed in a stereotaxic frame and a midline scalp incision was made to expose the skull. A guide cannula (coordinates from Bregma; AP = -0.94 mm, L = − 2.85 mm) and three electrodes, one on top of each hippocampus and the reference electrode on top of the frontal cortex, were fixed using dental cement to record surface electroencephalograms (EEG). Status epilepticus was induced by a microinjection of 0.2 µg kainic acid (KA) in 0.2 µl phosphate-buffered saline (PBS, Sigma-Aldrich, Dublin, Ireland) into the right basolateral amygdala. 40 min following intraamygdala KA injection, Lorazepam (6 mg/kg) (Wyeth, Taplow, UK) was delivered intraperitoneally to curtail seizures and reduce morbidity and mortality. EEG was recorded using a Xltek EEG system (Optima Medical Ltd., Guildford, UK) and recordings were commenced prior to intraamygdala KA injection and continued for 1 h post-lorazepam. EEG recordings were analysed by uploading EEG onto Labchart 8 reader software (ADInstruments) and total seizure power of EEG signals was calculated^20^.

#### Fluoro-Jade B staining

To assess status epilepticus-induced neurodegeneration, Fluoro-Jade B (FjB) staining was carried out as before^51^. Briefly, 12 μm coronal sections at the medial level of the hippocampus (Bregma AP = − 1.94 mm) were cut on a cryostat. Tissue was fixed in 4% PFA, rehydrated in ethanol, and then transferred to a 0.006% potassium permanganate solution followed by incubation with 0.001% FjB (Chemicon Europe Ltd, Chandlers Ford, UK). Sections were mounted in Dibutylphthalate Polystyrene Xylene (DPX) mounting solution (Sigma Aldrich, Dublin, Ireland). Using an epifluorescence microscope, cells including all hippocampal subfields (dentate gyrus (DG), cornu amonis regions 1 and 3 (CA1 and CA3)) were counted under a 40 × lens in two adjacent sections and the average determined for each animal.

#### Statistical analysis

GraphPad Prism software was used to perform statistical analysis and data were presented as means ± standard error of the mean (SEM). Student’s t-test was used to determine statistical differences between groups. Significance was accepted at *p < 0.05, **p < 0.01 ***s < 0.001.

## Supplementary information

Supplementary Information.

## References

[1] Di Virgilio F, Sarti AC, Grassi F (2018). Modulation of innate and adaptive immunity by P2X ion channels. Curr. Opin. Immunol..

[2] Kanellopoulos JM, Delarasse C (2019). Pleiotropic roles of P2X7 in the central nervous system. Front. Cell Neurosci..

[3] Beamer E, Fischer W, Engel T (2017). The ATP-gated P2X7 receptor as a target for the treatment of drug-resistant epilepsy. Front. Neurosci..

[4] Biber K (2019). Microglial drug targets in ad: opportunities and challenges in drug discovery and development. Front. Pharmacol..

[5] Deussing JM, Arzt E (2018). P2X7 receptor: a potential therapeutic target for depression?. Trends Mol. Med..

[6] Domercq M, Matute C (2019). Targeting P2X4 and P2X7 receptors in multiple sclerosis. Curr. Opin. Pharmacol..

[7] Koch-Nolte F (2019). Novel biologics targeting the P2X7 ion channel. Curr. Opin. Pharmacol..

[8] Diaz-Hernandez M (2009). Altered P2X7-receptor level and function in mouse models of Huntington's disease and therapeutic efficacy of antagonist administration. FASEB J..

[9] Marin-Garcia P, Sanchez-Nogueiro J, Gomez-Villafuertes R, Leon D, Miras-Portugal MT (2008). Synaptic terminals from mice midbrain exhibit functional P2X7 receptor. Neuroscience.

[10] Ohishi A (2016). Expression level of P2X7 receptor is a determinant of ATP-induced death of mouse cultured neurons. Neuroscience.

[11] Illes P, Khan TM, Rubini P (2017). Neuronal P2X7 receptors revisited: do they really exist?. J. Neurosci..

[12] Miras-Portugal MT, Sebastián-Serrano Á, de Diego García L, Díaz-Hernández M (2017). Neuronal P2X7 receptor: involvement in neuronal physiology and pathology. The Journal of Neuroscience.

[13] Gong S (2003). A gene expression atlas of the central nervous system based on bacterial artificial chromosomes. Nature.

[14] Heintz N (2001). BAC to the future: the use of bac transgenic mice for neuroscience research. Nat. Rev. Neurosci..

[15] Yang, X. W. & Gong, S. An overview on the generation of BAC transgenic mice for neuroscience research. *Curr. Protocols Neurosci.* **Chapter 5**, Unit 5.20. 10.1002/0471142301.ns0520s31 (2005). 10.1002/0471142301.ns0520s31 18428622

[16] Gerfen CR, Paletzki R, Heintz N (2013). GENSAT BAC cre-recombinase driver lines to study the functional organization of cerebral cortical and basal ganglia circuits. Neuron.

[17] Srinivasan R (2016). New transgenic mouse lines for selectively targeting astrocytes and studying calcium signals in astrocyte processes in situ and in vivo. Neuron.

[18] Xu J (2016). P2X4 receptor reporter mice: sparse brain expression and feeding-related presynaptic facilitation in the arcuate nucleus. J. Neurosci..

[19] Gong S, Kus L, Heintz N (2010). Rapid bacterial artificial chromosome modification for large-scale mouse transgenesis. Nat. Protoc..

[20] Engel T (2012). Seizure suppression and neuroprotection by targeting the purinergic P2X7 receptor during status epilepticus in mice. FASEB J..

[21] Garcia-Huerta P (2012). The specificity protein factor Sp1 mediates transcriptional regulation of P2X7 receptors in the nervous system. J. Biol. Chem..

[22] Hirayama Y (2015). Astrocyte-mediated ischemic tolerance. J. Neurosci..

[23] Martinez-Frailes C (2019). Amyloid peptide induced neuroinflammation increases the P2X7 receptor expression in microglial cells, impacting on its functionality. Front. Cell Neurosci..

[24] Kaczmarek-Hajek, K. *et al.* Re-evaluation of neuronal P2X7 expression using novel mouse models and a P2X7-specific nanobody. *eLife 7, e36217*. 10.7554/elife.36217 (2018). 10.7554/eLife.36217 PMC6140716 30074479

[25] Perrault I (2015). IFT81, encoding an IFT-B core protein, as a very rare cause of a ciliopathy phenotype. J. Med. Genet..

[26] Wachter S (2019). Binding of IFT22 to the intraflagellar transport complex is essential for flagellum assembly. EMBO J..

[27] Collo G (1996). Cloning OF P2X5 and P2X6 receptors and the distribution and properties of an extended family of ATP-gated ion channels. J. Neurosci..

[28] Soto F (1996). P2X4: an ATP-activated ionotropic receptor cloned from rat brain. Proc. Natl. Acad. Sci. USA.

[29] Bertin E (2020). Increased surface P2X4 receptor regulates anxiety and memory in P2X4 internalization-defective knock-in mice. Mol. Psychiatry.

[30] Murrell-Lagnado RD, Frick M (2019). P2X4 and lysosome fusion. Curr. Opin. Pharmacol..

[31] Qureshi OS, Paramasivam A, Yu JCH, Murrell-Lagnado RD (2007). Regulation of P2X4 receptors by lysosomal targeting, glycan protection and exocytosis. J. Cell Sci..

[32] Varga RE (2015). In vivo evidence for lysosome depletion and impaired autophagic clearance in hereditary spastic paraplegia type SPG11. PLoS Genet..

[33] Nicke A (2009). A functional P2X7 splice variant with an alternative transmembrane domain 1 escapes gene inactivation in P2X7 knock-out mice. J. Biol. Chem..

[34] Jimenez-Pacheco A (2013). Increased neocortical expression of the P2X7 receptor after status epilepticus and anticonvulsant effect of P2X7 receptor antagonist A-438079. Epilepsia.

[35] Ulmann L (2013). Involvement of P2X4 receptors in hippocampal microglial activation after status epilepticus. Glia.

[36] Mouri G (2008). Unilateral hippocampal CA3-predominant damage and short latency epileptogenesis after intra-amygdala microinjection of kainic acid in mice. Brain Res..

[37] Jimenez-Pacheco A (2016). Transient P2X7 receptor antagonism produces lasting reductions in spontaneous seizures and gliosis in experimental temporal lobe epilepsy. J. Neurosci..

[38] Shuen JA, Chen M, Gloss B, Calakos N (2008). Drd1a-tdTomato BAC transgenic mice for simultaneous visualization of medium spiny neurons in the direct and indirect pathways of the basal ganglia. J. Neurosci..

[39] Schmidt EF, Kus L, Gong S, Heintz N (2013). BAC transgenic mice and the GENSAT database of engineered mouse strains. Cold Spring Harbor Protoc..

[40] Chen Y, Molet J, Gunn BG, Ressler K, Baram TZ (2015). Diversity of reporter expression patterns in transgenic mouse lines targeting corticotropin-releasing hormone-expressing neurons. Endocrinology.

[41] Zhao XF (2019). Targeting microglia using Cx3cr1-Cre lines: revisiting the specificity. eNeuro.

[42] Beil J, Fairbairn L, Pelczar P, Buch T (2012). Is BAC transgenesis obsolete? State of the art in the era of designer nucleases. J. Biomed. Biotechnol..

[43] Kopp R, Krautloher A, Ramirez-Fernandez A, Nicke A (2019). P2X7 interactions and signalling: making head or tail of it. Front. Mol. Neurosci..

[44] Srivastava PK (2017). Genome-wide analysis of differential RNA editing in epilepsy. Genome Res..

[45] Weill L, Belloc E, Bava FA, Mendez R (2012). Translational control by changes in poly(A) tail length: recycling mRNAs. Nat. Struct. Mol. Biol..

[46] Jimenez-Mateos EM (2015). microRNA targeting of the P2X7 purinoceptor opposes a contralateral epileptogenic focus in the hippocampus. Sci. Rep..

[47] Engel T (2013). CHOP regulates the p53-MDM2 axis and is required for neuronal survival after seizures. Brain.

[48] Refojo D (2011). Glutamatergic and dopaminergic neurons mediate anxiogenic and anxiolytic effects of CRHR1. Science.

[49] Zhang J (2013). Germ-line recombination activity of the widely used hGFAP-Cre and nestin-Cre transgenes. PLoS ONE.

[50] Rissiek B (2020). Astrocytes and microglia are resistant to NAD(+)-mediated cell death along the ARTC2/P2X7 axis. Front. Mol. Neurosci..

[51] Engel T (2018). Bi-directional genetic modulation of GSK-3beta exacerbates hippocampal neuropathology in experimental status epilepticus. Cell Death Dis..

