# Supplementary material for: Deviant reporter expression and P2X4 passenger gene overexpression in the soluble EGFP BAC transgenic P2X7 reporter mouse model
Source: Sci Rep. 2020 Nov 16;10:19876. doi: 10.1038/s41598-020-76428-0 (PMC7669894; doi:10.1038/s41598-020-76428-0)
Supplement: Supplementary file 1 — Supplementary Information. [file 41598_2020_76428_MOESM1_ESM.pdf]

Deviant reporter expression and P2X4 passenger gene overexpression in the soluble EGFP BAC transgenic P2X7 reporter mouse model

Antonio Ramírez-Fernández[1], Lidia Urbina-Treviño[2], Giorgia Conte[3], Mariana Alves[3], Björn Rissiek[4], Anna Durner[1], Nicolas Scalbert[1], Jiong Zhang[1], Tim Magnus[4], Friedrich Koch-Nolte[5], Nikolaus Plesnila[6], Jan M. Deussing[2], Tobias Engel[3,7], Robin Kopp[1], Annette Nicke[1]*

[1] Walther Straub Institute of Pharmacology and Toxicology, Faculty of Medicine, LMU Munich, Munich, Germany
[2] Max Planck Institute of Psychiatry, Molecular Neurogenetics, Munich, Germany
[3] Department of Physiology & Medical Physics, Royal College of Surgeons in Ireland, Dublin D02 YN77, Ireland
[4] Department of Neurology, University Medical Center Hamburg-Eppendorf, Hamburg, Germany
[5] Institute of Immunology, University Medical Center Hamburg-Eppendorf, Hamburg, Germany
[6] Institute for Stroke and Dementia Research, Munich University Hospital, LMU Munich, Germany
[7] FutureNeuro, SFI Research Centre for Chronic and Rare Neurological Diseases. RCSI, Dublin D02 YN77, Ireland

Correspondence:
Annette Nicke
annette.nicke@lrz.uni-muenchen.de

Running title: BAC transgene comparison

Suppl. Fig. 1

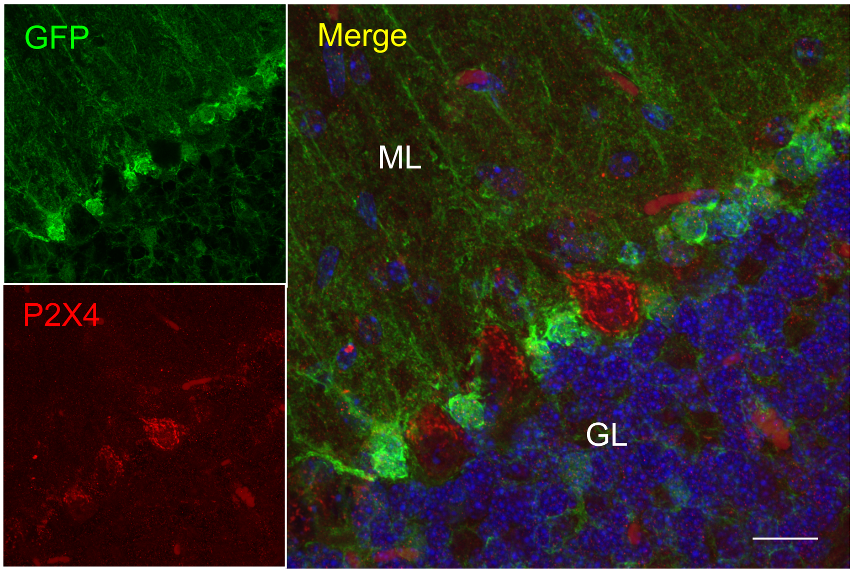

Supplementary Figure 1:
P2X4 Expression in Purkinje cells of the sEGFP mouse model.
Immunofluorescence staining of cerebellar slices of the sEGFP mouse with antibodies against P2X4 and GFP (Thermo Fischer CA10262). Nuclear staining with DAPI is shown in blue. A representative image out of the three individual animals is shown. GL, Granular cell layer; ML, molecular cell layer. Scale bar: 20 µm.

Suppl. Fig. 2

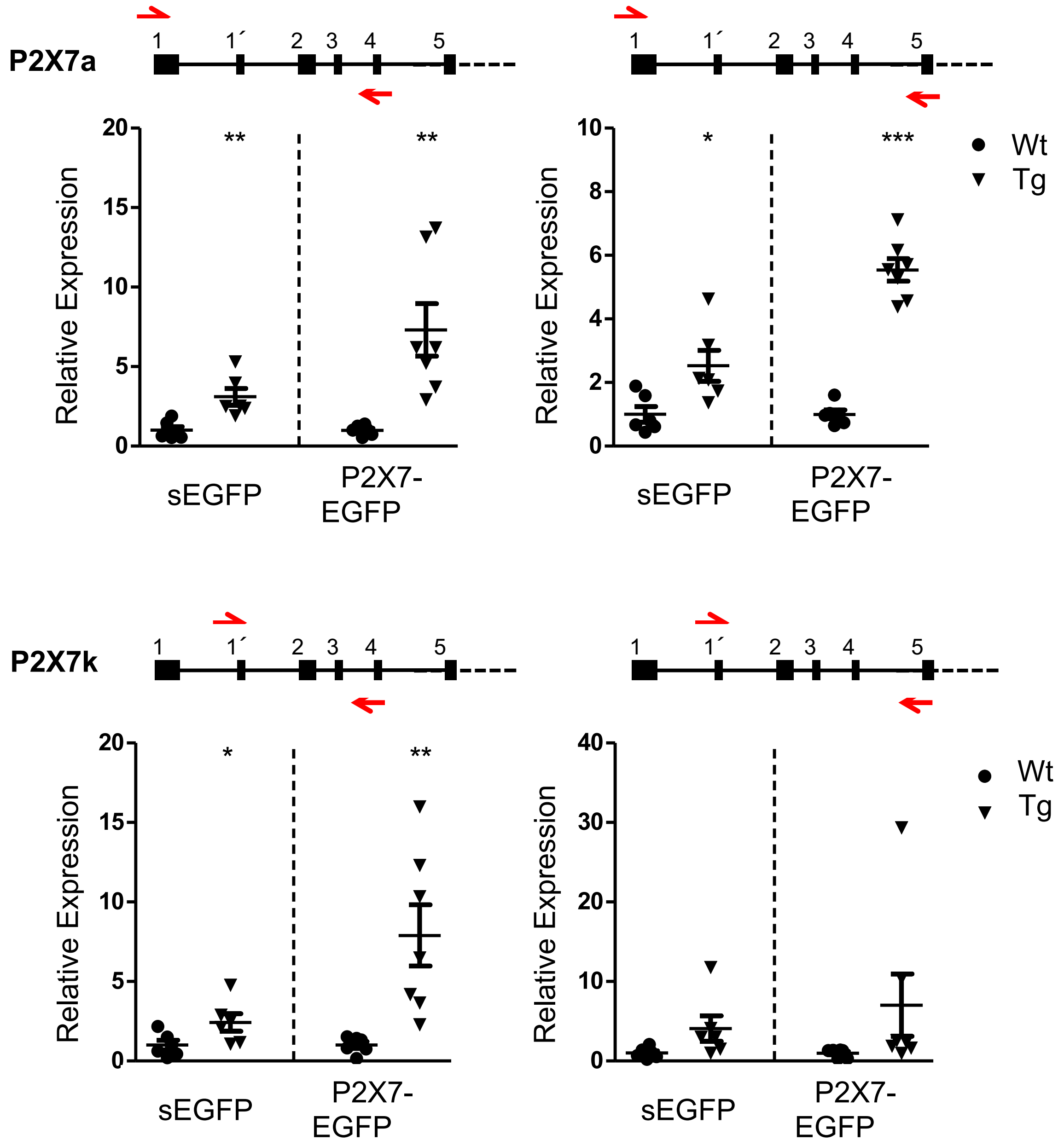

Supplementary Figure 2:

P2X7 Splice variant expression in sEGFP and P2X7-EGFP transgenic models. RNA was extracted from hippocampus and quantitative PCR was performed with primers directed against sequences in the indicated *P2rx7* exons. Data were normalized to expression of β-actin and the respective levels in wt controls. Bars represent mean ± SEM from 2 independent experiments and 6-7 mice. Significance was analysed using unpaired two-tailed Student's t-test and is indicated as *p<0.05, **p<0.01, ***p<0.001 (*P2rx7a* levels Ex1-Ex4: sEGFP 3.105 ± 0.5231, n=6; P2X7-EGFP 7.309 ± 1.649, n=7, Ex1-Ex5: sEGFP 2.525 ± 0.4881, n=6; P2X7-EGFP 5.541 ± 0.3531, n=7; *P2rx7k* levels Ex1'-Ex4 sEGFP 2.425 ± 0.5552, n=6, P2X7-EGFP 7.897 ± 1.926, n=7; Ex1'-Ex5 sEGFP 4.087 ± 1.606, n=6; P2X7-EGFP 7.024 ± 3.911, n=7).

Suppl. Fig. 3

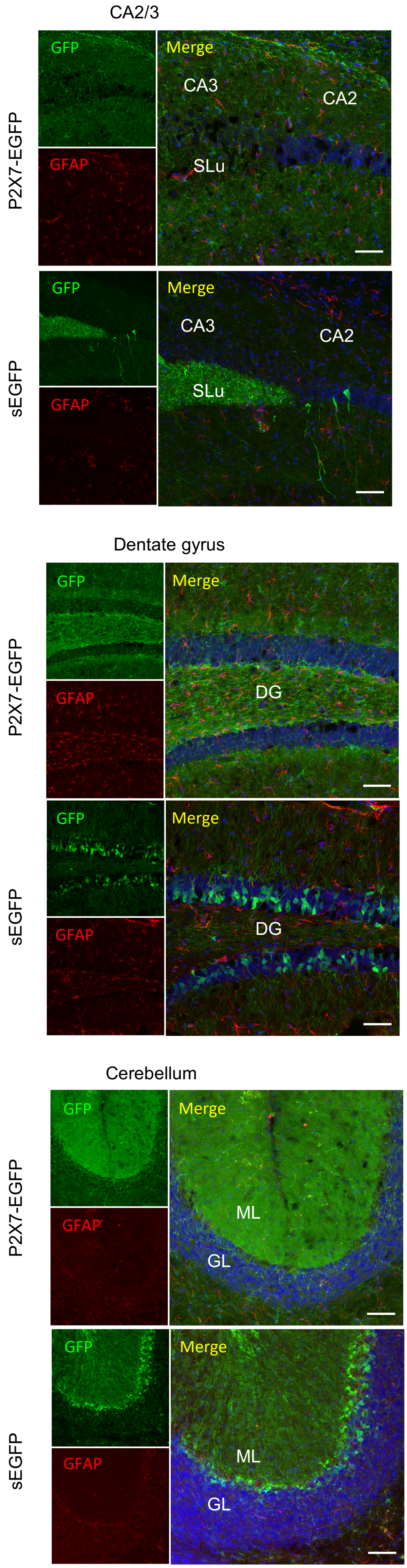

Supplementary Figure 3:
Analysis on the colocalization of EGFP with the astrocytic marker GFAP in sEGFP and P2X7-EGFP BAC transgenic P2X7 reporter mice.
Saggital brain slices (40 µm) were labelled with antibodies against GFAP and GFP (Abcam ab6556). Images show representative samples from at least three mice per group in the following regions: (**A**) CA3-CA2 border (**B**) Dentate gyrus (**C**) Cerebellum. DAPI staining is shown in blue. Scale bars: 50 µm.

Suppl. Fig. 4

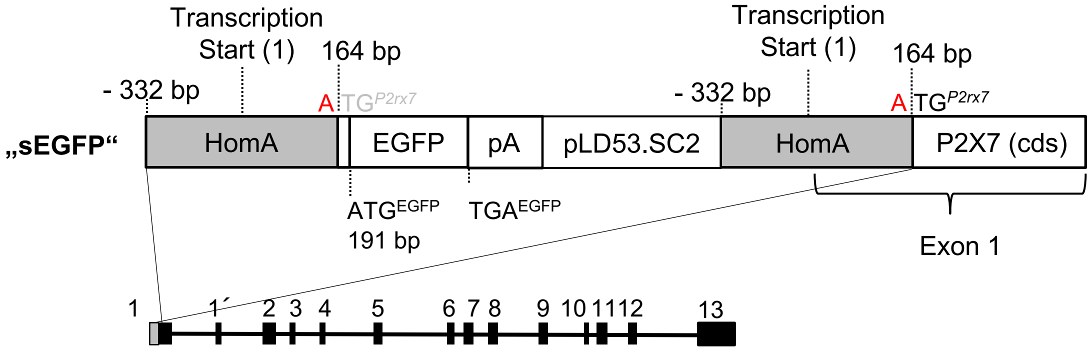

Supplementary Figure 4:
Scheme depicting the integration of the targeting vector encoding the soluble EGFP into the Start ATG of the *P2rx7* gene.
The A of the *P2rx7* Start ATG is the last residue in the Homology arm (Hom A) and shown in red. The bases TG (shown in grey) are replaced by 27 bp of targeting vector sequence followed by the EGFP coding sequence, poly adenylation signal and the targeting vector pLD53.SC2. Due to the non-resolving homologous recombination strategy, the homology region A is duplicated and therefore complements the *P2rx7* Start ATG.

Suppl. Fig. 5

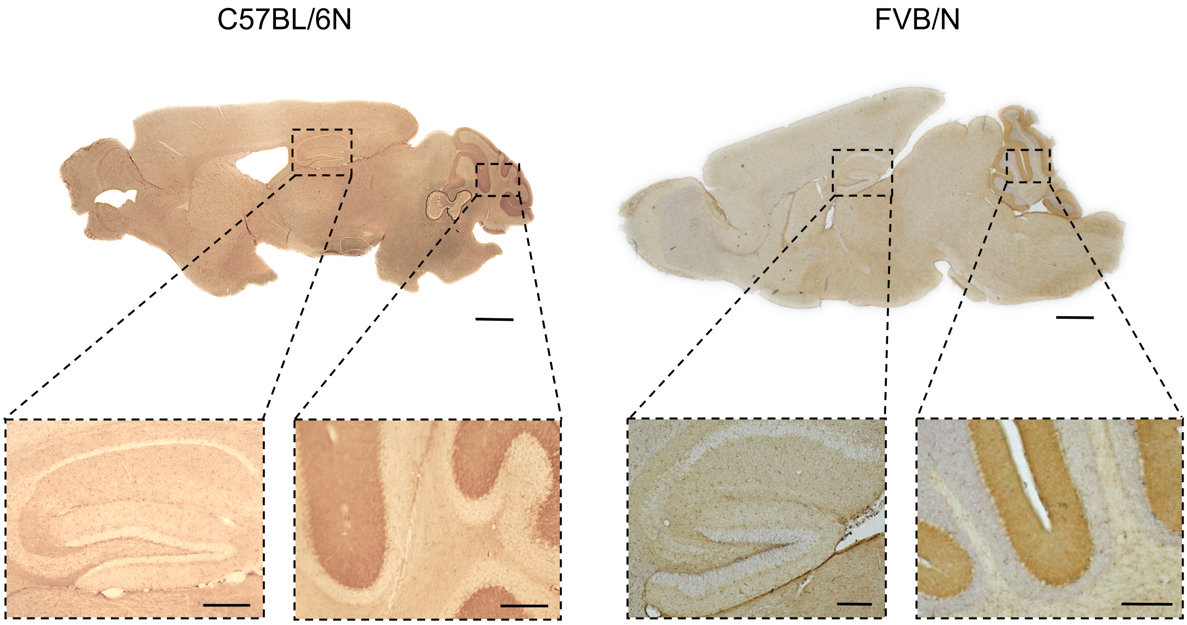

Supplementary Figure 5:
Direct comparison of P2X7 protein expression pattern in wildtype mice from C57BL/6N and FVB/N genetic backgrounds. DAB staining was performed with a P2X7-specific nanobody. Note that no counterstaining for haematoxylin was performed in the presented C57BL/6 sample and that images were taken on different microscopes. Scale bars: 200 µm.

Suppl. Fig. 6

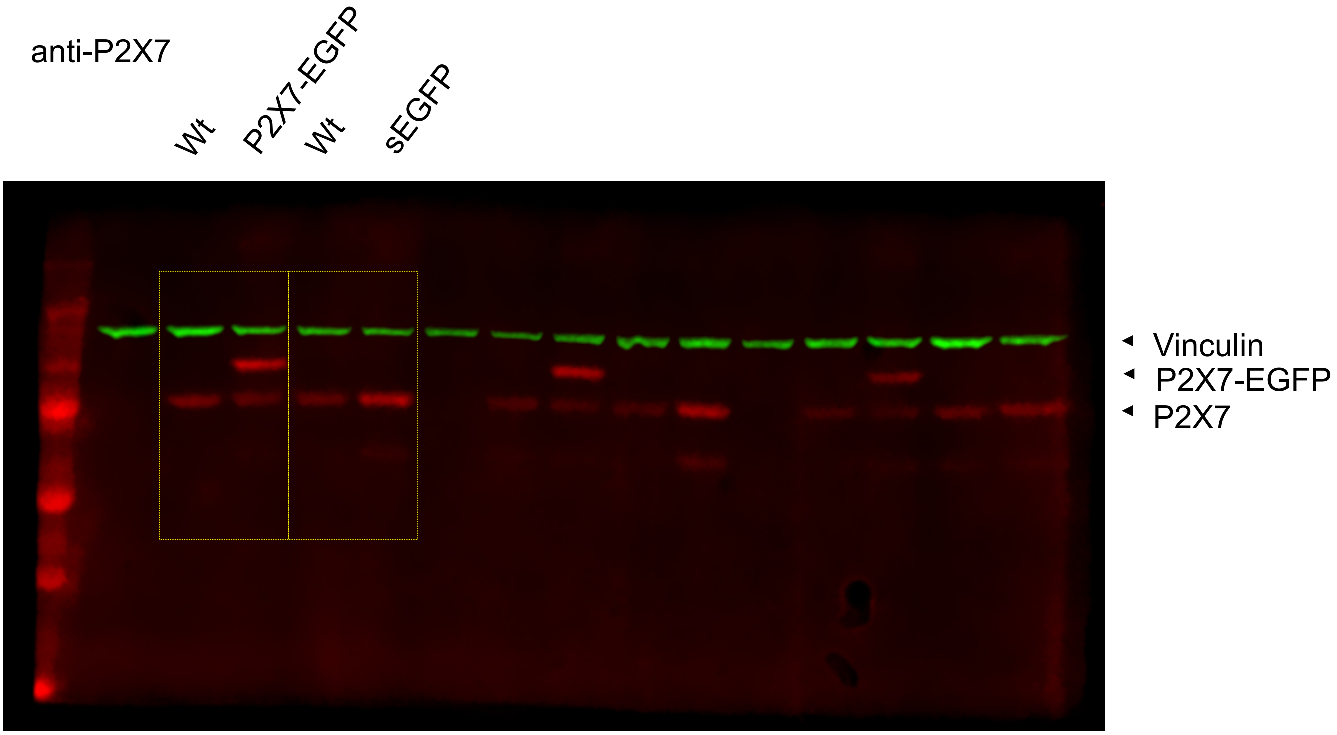

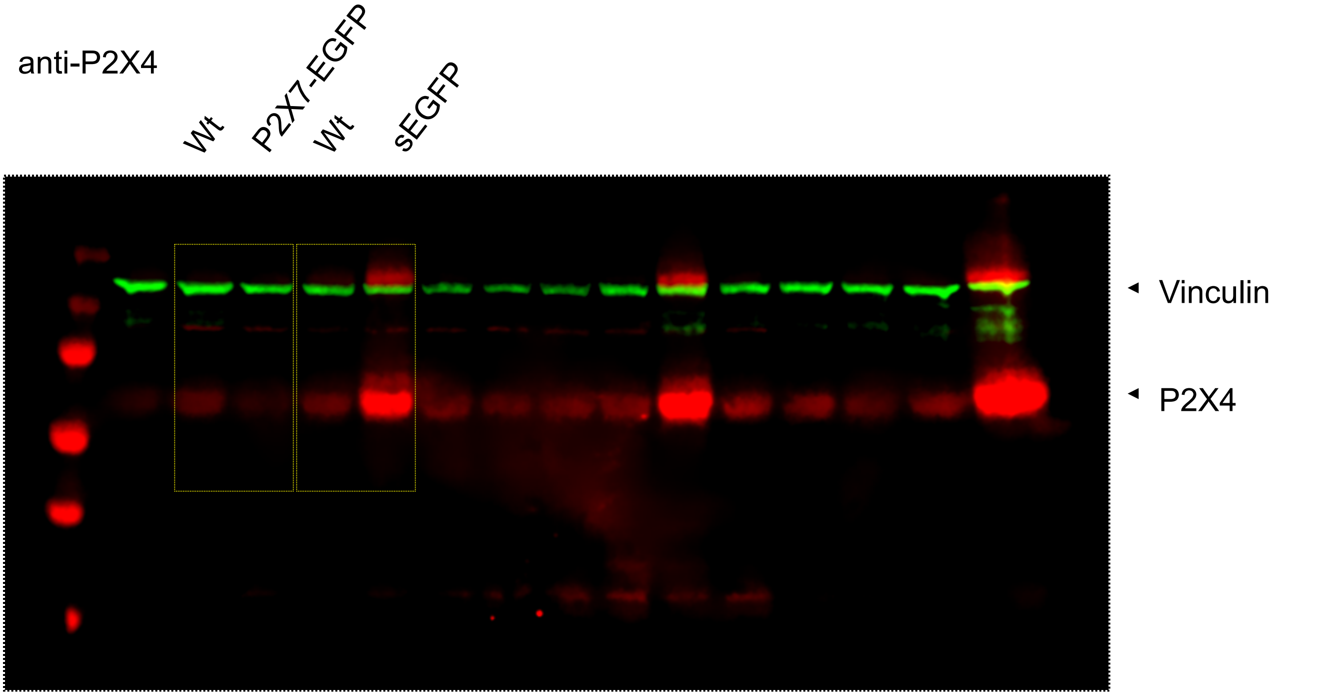

Supplementary Figure 6:
Full-length blots from Figure 1C.
The parts shown in Fig 1C are indicated by dashed lines. For details see Figure 1C.

Supplementary Table 1:
Antibodies used for Western blotting and immunohistochemistry

Antibody	Supplier	Cat#<br>RRID:	Application and dilution
P2X7 C-term (rb pAb)	Synaptic Systems	177003,<br>AB_887755	WB 1:1500
P2X4	Alomone	APR-002,<br>AB_2040058	WB 1:1000<br>IHC 1:200
Vinculin<br>(ms hVin-1)	Sigma-Aldrich	V9131,<br>AB_477629	WB 1:10.000
800CW gt anti-ms	LI-COR	925–32210,<br>AB_2687825	WB 1:15.000
680RD dk anti-rb	LI-COR	925–68073,<br>AB_2716687	WB 1:15.000
P2X7 ECD, 7E2-rbIgG	Nolte lab	Nanobody rbIgG fusion construct	DAB 6.7 ng/mL
GFP (rb pAb)	Abcam	ab6556,<br>AB_305564	IHC 1:2000<br>DAB 1:5000
GFP<br>(chk pAb)	Thermo Fisher	CA10262,<br>AB_2534023	IHC 1:400
GFP<br>(gt pAb)	Abcam	AB_5450<br>AB_304897	IHC 1:100
NeuN<br>(ms A60)	Millipore	MAB377,<br>AB_2298772	IHC 1:500
GFAP<br>(ms GA5)	Millipore/<br>Sigma-Aldrich	MAB360,<br>AB_11212597	IHC 1:200
Iba1<br>(rb pAb)	WAKO	019–19741,<br>AB_839504	IHC 1:100
Olig 2<br>(ms 211F1.1)	Millipore	MABN50,<br>AB_10807410	IHC 1:200
Calbindin D28k<br>(ms CB-955)	Sigma-Aldrich	C9848, AB_476894	IHC 1:1000
Calretinin<br>(ms 37C9)	Synaptic Systems	214111,<br>AB_2619904	IHC 1:200
Parvalbumin (ms 58E1)	Synaptic Systems	195011,<br>AB_2619882	IHC 1:500
ZnT3<br>(ms 180C1)	Synaptic Systems	197011,<br>AB_2189665	IHC 1:100
S100β<br>(rb pAb)	Synaptic Systems	287003,<br>AB_2620024	IHC 1:500
Collagen IV<br>(rb pAb)	Abcam	AB_19808<br>AB_445160	IHC 1:100
CD 206<br>(rb mAb)	Biorad	MCA2235GA<br>AB_322613	IHC 1:100
A594<br>gt anti-rb	Thermo Fisher	A11037,<br>AB_2534095	IHC 1:400
A594<br>gt anti-ms	Thermo Fisher	A11032,<br>AB_2534091	IHC 1:400
A594<br>gt anti-rat	Thermo Fisher	A11007,<br>AB_10561522	IHC 1:400
A546<br>gt anti-ms	Thermo Fisher	A-11003,<br>AB_2534071	IHC 1:400
A488<br>gt anti-rb	Thermo Fisher	A11008,<br>AB_143165	IHC 1:400
A488<br>gt anti-chk	Thermo Fisher	A11039,<br>AB_2534096	IHC 1:400
